# Primary Extrahepatic Biliary Mucinous Cystic Neoplasm Suspected Preoperatively and Treated by Robotic Pancreaticoduodenectomy: A Case Report

**DOI:** 10.70352/scrj.cr.26-0068

**Published:** 2026-05-19

**Authors:** Yukihiro Shimogata, Kazuharu Igarashi, Hiroshi Tajima, Tomoyuki Kato, Toshimasa Fujio, Yoshiki Fujiyama, Nobuyuki Nishizawa, Masafumi Watanabe, Kosuke Okuwaki, Masataka Tochimoto, Takafumi Sangai, Naoki Hiki, Takeshi Naitoh, Yusuke Kumamoto

**Affiliations:** 1 Department of General-Pediatric Hepatobiliary Pancreatic Surgery, Kitasato University School of Medicine, Sagamihara, Kanagawa, Japan; 2 Department of Gastroenterology, Kitasato University School of Medicine, Sagamihara, Kanagawa, Japan; 3 Department of Pathology, Kitasato University School of Medicine, Sagamihara, Kanagawa, Japan; 4 Department of Breast and Thyroid Surgery, Kitasato University School of Medicine, Sagamihara, Kanagawa, Japan; 5 Department of Upper Gastrointestinal Surgery, Kitasato University School of Medicine, Sagamihara, Kanagawa, Japan; 6 Department of Lower Gastrointestinal Surgery, Kitasato University School of Medicine, Sagamihara, Kanagawa, Japan

**Keywords:** biliary mucinous cystic neoplasm, biliary cystadenoma, extrahepatic bile duct, endoscopic ultrasound, robotic pancreaticoduodenectomy, preoperative suspicion, ovarian-like stroma

## Abstract

**INTRODUCTION:**

Biliary mucinous cystic neoplasm (MCN) is a cystic tumor characterized by ovarian-like stroma and is recognized as a neoplasm with the potential for invasive carcinoma. While MCNs predominantly occur in the pancreas, primary occurrence in the extrahepatic bile duct is exceedingly rare. Most reported cases are diagnosed postoperatively due to the lack of specific clinical symptoms, the difficulty in preoperative differentiation from other cystic diseases, such as intraductal papillary neoplasm of the bile duct, and the strict requirement of pathological evaluation for definitive diagnosis. Here, we report a case of primary extrahepatic biliary MCN that was strongly suspected preoperatively using multimodal imaging and treated with robotic surgery.

**CASE PRESENTATION:**

A 48-year-old woman presented to a previous hospital with jaundice and abdominal pain. CT revealed a 40-mm cystic lesion in the common bile duct. Despite biliary stenting for obstructive jaundice, she experienced recurrent cholangitis and was referred to our hospital for further investigation and treatment. Detailed imaging evaluation, including endoscopic ultrasound (EUS), identified characteristic findings of MCN, such as a thick capsule and a cyst-in-cyst appearance. The absence of continuity with the pancreas led to the preoperative clinical suspicion of MCN originating from the bile duct wall. As the tumor extended into the intrapancreatic bile duct and was suspected to be benign or low-grade, we performed robotic pylorus-preserving pancreaticoduodenectomy aiming to balance radicality with minimal invasiveness. Histopathology confirmed primary extrahepatic biliary MCN with low-grade dysplasia. The patient was discharged on POD 8 and remains recurrence-free at 6 months’ follow-up.

**CONCLUSIONS:**

Although primary extrahepatic biliary MCN is an extremely rare entity, this case suggests that detailed multimodal imaging, particularly EUS, may help with preoperative suspicion of biliary MCN by identifying characteristic features. The characteristic findings presented herein will serve as a valuable reference for the preoperative differentiation of this rare disease. While complete surgical resection is mandatory, our experience suggests that robotic surgery can serve as a minimally invasive approach capable of achieving this. To the best of our knowledge, this is the first reported case of primary extrahepatic biliary MCN treated by robotic surgery, representing a feasible therapeutic option.

## Abbreviations

ER
: estrogen receptor
ERBD
: endoscopic retrograde biliary drainage
ERCP
: endoscopic retrograde cholangiopancreatography
EUS
: endoscopic ultrasound
IPNB
: intraductal papillary neoplasm of the bile duct
MCN
: mucinous cystic neoplasm
MRCP
: magnetic resonance cholangiopancreatography
OLS
: ovarian-like stroma
PD
: pancreaticoduodenectomy
PR
: progesterone receptor
POCS
: peroral cholangioscopy
RPPPD
: robotic pylorus-preserving pancreaticoduodenectomy
WHO
: World Health Organization

## INTRODUCTION

Biliary MCN is a cyst-forming epithelial neoplasm and is recognized as a neoplasm with potential for invasive carcinoma, defined by the presence of OLS according to the current WHO Classification.^1)^ While MCNs primarily occur in the pancreas, they rarely arise in the biliary system, and primary extrahepatic biliary MCNs are exceedingly rare. In addition to its rarity, primary extrahepatic biliary MCN is difficult to differentiate from other cystic diseases such as IPNB. Furthermore, because a definitive diagnosis strictly requires the histopathological identification of OLS, establishing a preoperative definitive diagnosis is extremely challenging.^2)^ Given the potential for malignant transformation and high recurrence rate, complete surgical resection is the standard treatment.^3,4)^

We report a case of primary extrahepatic biliary MCN that was strongly suspected preoperatively by detailed imaging and treated with radical resection by RPPPD, along with a review of the literature.

## CASE PRESENTATION

**Fig. 1:**
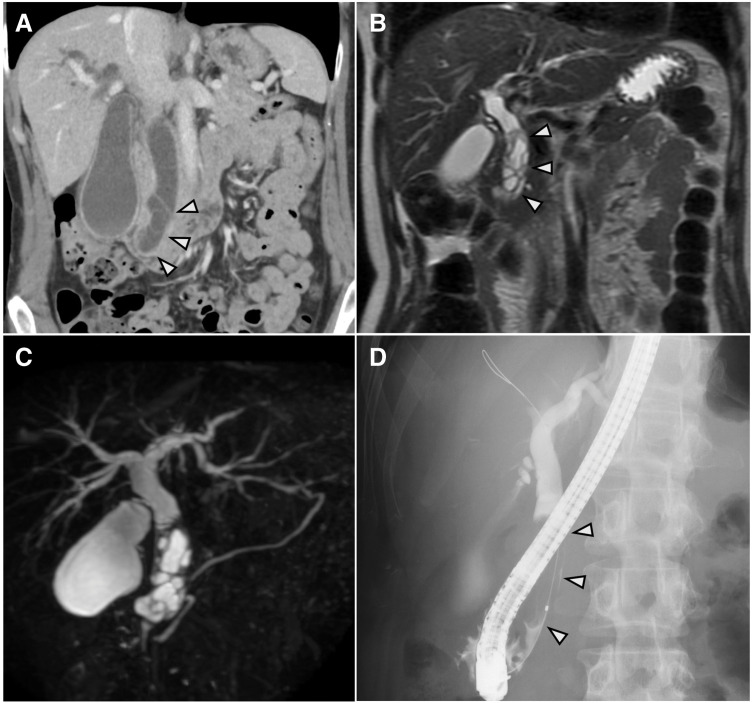
Preoperative imaging findings. (**A**) Abdominal contrast-enhanced CT (coronal view) during the portal venous phase at the initial visit to the previous hospital: A multilocular cystic lesion is observed in the common bile duct (arrowheads). (**B**) MRI T2-weighted image (coronal view): A multilocular cystic tumor showing high signal intensity is observed in the common bile duct. The tumor is covered by a thick low-signal capsule, and multiple internal septal structures can be confirmed (arrowheads). (**C**) MRCP: No anatomical anomalies of the bile duct or pancreaticobiliary maljunction are observed. (**D**) ERCP: A filling defect is observed in the common bile duct. There is no communication between the tumor and the bile duct (arrowheads). ERCP, endoscopic retrograde cholangiopancreatography; MRCP, magnetic resonance cholangiopancreatography

A 48-year-old woman with an unremarkable past medical history presented to a previous hospital with jaundice noted 1 month prior, followed by abdominal pain. Blood tests revealed obstructive jaundice (total bilirubin 10.0 mg/dL, direct bilirubin 7.3 mg/dL) and elevated hepatobiliary enzymes (aspartate aminotransferase [AST] 93 U/L, alanine aminotransferase [ALT] 86 U/L, alkaline phosphatase [ALP] 345 U/L, γ-glutamyl transferase [γ-GTP] 127 U/L). CT showed a cystic mass measuring 40 mm in maximum diameter in the common bile duct with biliary dilation (**Fig. 1A**). ERCP was performed for suspected choledocholithiasis, but no stones were confirmed, and a plastic stent was placed for ERBD. Subsequently, she experienced 2 episodes of cholangitis despite drainage. Although a choledochal cyst was suspected, a definitive diagnosis remained elusive. Due to this diagnostic dilemma and recurrent cholangitis, she was referred to our hospital 4 months after the initial visit.

At the initial visit to our hospital, hepatobiliary enzymes had normalized following drainage (total bilirubin 0.8 mg/dL, direct bilirubin 0.2 mg/dL, AST 25 U/L, ALT 18 U/L, ALP 86 U/L, γ-GTP 93 U/L). Tumor markers were within normal limits (carcinoembryonic antigen 1.6 ng/mL, carbohydrate antigen 19-9 <3.0 U/mL).

**Fig. 2:**
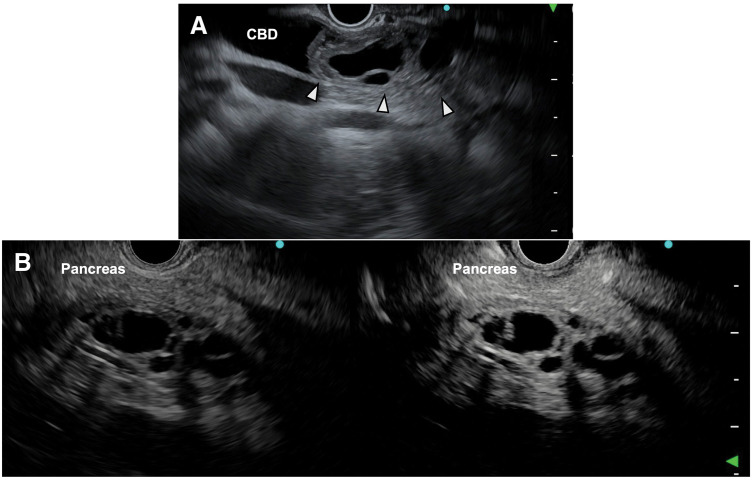
EUS imaging findings. (**A**) A multilocular cystic tumor covered by a thick capsule is confirmed in the CBD (arrowheads). The tumor arises from the bile duct wall. (**B**) Contrast-enhanced EUS with perflubutane shows multiple septa enhancing within the cyst, presenting a cyst-in-cyst appearance (left: pre-contrast; right: post-contrast). CBD, common bile duct, EUS, endoscopic ultrasound

Abdominal contrast-enhanced CT at our hospital revealed a 50-mm multilocular cystic tumor in the common bile duct, which showed a slight increase in size compared to the findings at the previous hospital. The cyst wall and internal septa showed contrast enhancement, and no obvious calcification or solid components were observed, which was similar to the previous CT appearance. MRI T2-weighted images showed a multilocular cystic tumor with high signal intensity in the common bile duct, covered by a thick low-signal capsule, with multiple internal septal structures (**Fig. 1B**). MRCP revealed no anatomical anomalies of the bile duct or pancreaticobiliary maljunction (**Fig. 1C**). ERCP showed a filling defect in the common bile duct, and no communication between the tumor and the bile duct was confirmed (**Fig. 1D**). Bile cytology was performed twice but was Class I. No mucus retention was observed in the bile duct. EUS confirmed a multilocular cystic tumor covered by a thick capsule in the common bile duct. There was no continuity between the tumor and the pancreas, suggesting its origin from the bile duct wall (**Fig. 2A**). Contrast-enhanced EUS with perflubutane showed multiple septa enhancing within the tumor, presenting a cyst-in-cyst appearance (**Fig. 2B**). No internal solid components or mural nodules suggestive of malignancy were observed within the cyst or on the wall.

Based on the clinical and imaging findings of a multilocular cystic tumor originating from the common bile duct in a middle-aged woman, presenting with a thick capsule and cyst-in-cyst appearance, MCN was strongly suspected. The main differential diagnosis, IPNB, was ruled out based on the absence of intraductal papillary growth on EUS, lack of tumor–bile duct communication on ERCP, and no evidence of mucin hypersecretion. Thus, a clinical suspicion of primary extrahepatic biliary MCN was established.

Regarding the treatment strategy, given the tumor’s enlargement tendency and recurrent cholangitis, surgical resection was considered essential. As the tumor extended distally into the intrapancreatic bile duct, obtaining a negative distal margin via isolated extrahepatic bile duct resection was considered anatomically impossible. Consequently, PD was deemed necessary to achieve complete oncological resection. Additionally, since the tumor was suspected to be benign or low-grade malignant, we selected RPPPD to minimize surgical invasiveness while ensuring the same level of radicality as open surgery.

Surgery was performed using the da Vinci Xi Surgical System (Intuitive Surgical, Sunnyvale, CA, USA). A 20-mm umbilical incision was initially made for the camera port. Upon exploration of the abdominal cavity, there was no evidence of tumor exposure on the serosal surface, liver metastasis, or peritoneal dissemination. No obvious enlargement of regional lymph nodes was observed. Regional lymph node dissection was limited to the lymphatic tissue around the hepatoduodenal ligament and common hepatic artery, as the tumor was suspected to be benign or low-grade malignant. Despite inflammatory adhesions in the hepatoduodenal ligament due to recurrent cholangitis, precise dissection was carefully performed to strictly adhere to key oncological principles: namely, preservation of the tumor capsule, avoidance of the spillage of bile or cyst contents, and achievement of an adequate surgical margin. Intraoperative US confirmed that the tumor extended to the intrapancreatic bile duct. No tumor extension to the hepatic duct bifurcation was observed. The pancreas was transected at the level of the left border of the portal vein. To minimize the risk of peritoneal seeding from bile spillage, the bile duct was clamped and transected distal to the bifurcation as the final step of the resection phase. The resected specimen was placed in a retrieval bag and extracted through the umbilical incision without extension. Intraoperative frozen section analysis of the proximal and distal bile duct margins and the pancreatic resection margin confirmed the absence of malignancy. Reconstruction was performed using the modified Child method via a retrocolic route, with the pancreaticojejunostomy and hepaticojejunostomy performed robotically. During the hepaticojejunostomy, the clamp on the bile duct was released, and bile was meticulously suctioned to prevent intraperitoneal contamination. The operative time was 9 hours and 42 minutes, with a blood loss of 70 mL.

**Fig. 3:**
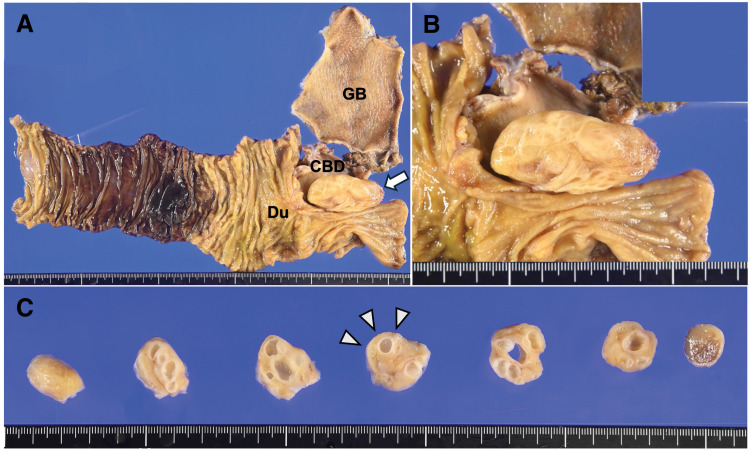
Macroscopic findings of the resected specimen. (**A**) Macroscopic view of the resected specimen: The anatomical relationships among the GB, CBD, and Du are shown. A 60 × 25 × 20-mm tumor is observed in the CBD (arrow). (**B**) Magnified view of the opened bile duct: The tumor arises from the bile duct wall. (**C**) Cut surface of the tumor: The tumor is covered by a thick fibrous capsule (arrowheads) and contains multilocular cysts of various sizes separated by septa, perfectly correlating with the “cyst-in-cyst” appearance observed on preoperative EUS. CBD, common bile duct; Du, duodenum; EUS, endoscopic ultrasound; GB, gallbladder

**Fig. 4:**
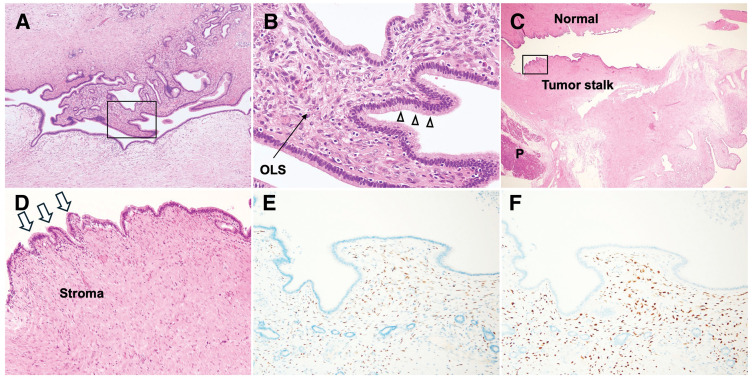
Histopathological and immunohistochemical findings. (**A**) Low-power view (HE, ×40): The tumor consists of a multilocular cystic structure lined by mucinous epithelium. The boxed area is shown at higher magnification in (**B**). (**B**) High-power view (HE, ×200): Higher magnification of the boxed area in (**A**). The cysts are lined by columnar mucinous epithelium showing nuclear stratification and intracellular mucin (arrowheads), indicative of neoplastic proliferation. Immediately beneath the epithelium, a dense layer of spindle cells is observed, consistent with OLS. (**C**) Transition from normal bile duct (HE, ×20): Low-power view showing the anatomical relationship among the adjacent pancreatic tissue (P), the normal bile duct wall (Normal), and the tumor stalk. While the main body of the polypoid tumor was detached during processing, the site of origin is clearly identified. The boxed area is shown in (**D**). (**D**) Transition from normal bile duct (HE, ×100): Higher magnification of the tumor stalk (boxed area in **C**). The epithelium exhibits neoplastic changes, characterized by columnar cells with intracellular mucin and nuclear stratification (arrowheads). However, the underlying stroma resembles that of the normal bile duct, not showing a fully developed OLS (Stroma). This admixture of neoplastic epithelium and normal-appearing stroma strongly indicates a transitional phase arising from the bile duct wall. (**E**, **F**) Immunohistochemistry (×100): The spindle cells of the OLS show diffuse nuclear positivity for ER (**E**) and PR (**F**). ER, estrogen receptor; HE, hematoxylin and eosin; OLS, ovarian-like stroma; PR, progesterone receptor

Macroscopically, a 60 × 25 × 20-mm tumor was observed in the common bile duct (**Fig. 3A** and **3B**). The cut surface showed a thick fibrous capsule containing multilocular cysts of various sizes separated by septa (**Fig. 3C**). Histologically, the tumor was lined by columnar mucinous epithelium, partially showing nuclear stratification, and was positive for Periodic acid-Schiff staining. Findings of OLS consisting of dense spindle cells were observed immediately beneath the epithelium. The epithelial cells exhibited low-grade dysplasia with minimal atypia, and no obvious invasive growth or high-grade dysplasia was observed (**Fig. 4A** and **4B**). Immunohistochemically, the spindle cells constituting the OLS showed diffuse positivity for ER and PR (**Fig. 4E** and **4F**). Notably, at the tumor stalk, a histological transition was identified: while the epithelium exhibited neoplastic changes, the underlying stroma did not show a fully developed OLS but rather resembled the normal bile duct stroma. This finding strongly confirmed the tumor as a primary neoplasm arising from the bile duct wall (**Fig. 4C** and **4D**). Based on these findings, the definitive diagnosis of primary extrahepatic biliary MCN with low-grade dysplasia was made. The resection margin was negative, and no lymph node metastasis was observed. The postoperative course was uneventful, and the patient was discharged on POD 8. She remains recurrence-free at 6 months after surgery.

## DISCUSSION

Biliary MCN is a rare tumor previously known as “biliary cystadenoma” and “biliary cystadenocarcinoma.” The WHO Classification reorganized these conventional terms in 2010, and the current 2019 edition defines biliary MCN as “a cyst-forming epithelial neoplasm, typically showing no communication with the bile ducts, composed of cuboidal to columnar, variably mucin-producing epithelium, associated with ovarian-type subepithelial stroma”.^1)^ This stroma is clinically referred to as OLS, consisting of dense spindle cells similar to ovarian stroma and characterized by positive immunohistochemistry for ER and PR. Crucially, a definitive diagnosis requires the histopathological identification of OLS and cannot be established based on clinical or imaging findings alone. Due to this strict definition, it has been pointed out that cases previously reported as “biliary cystadenoma” might have included lesions lacking OLS, which should now be classified primarily as the cystic variant of IPNB.^2)^ Biliary MCNs are quite rare, with an estimated incidence of 1 case per 20000–100000 person-years.^1)^ Approximately 90%–95% of biliary MCNs occur in the intrahepatic bile ducts, and primary extrahepatic cases like the present one are extremely rare, accounting for only about 5%–10% of all biliary MCNs.^2,5)^ Biliary MCN occurs almost exclusively in middle-aged women, with a reported mean age at diagnosis of 51 years.^1)^ This marked gender difference is thought to be related to hormone dependence, suggested by the hormone receptor positivity of OLS. Indeed, cases of tumor enlargement during oral contraceptive use or pregnancy have been reported, supporting this hypothesis.^2,5)^

We searched PubMed from 2000 to 2025 using the terms “biliary MCN,” “biliary cystadenoma,” and “extrahepatic bile duct.” We identified 14 reported cases^4,6–18)^ that clearly originated from the extrahepatic bile duct and had pathologically confirmed OLS or hormone receptor (ER/PR) positivity, resulting in a total of 15 cases including the present case (**Table 1**). Note that 1 case was included because ER positivity was described, although OLS was not explicitly stated.^16)^ All patients were female, with a median age of 48 years (range: 24–62 years). Clinical symptoms frequently included abdominal pain and jaundice. The median tumor size was 41 mm (range: 18–100 mm).

**Table 1:** Summary of reported cases of primary extrahepatic biliary MCN

Case	Year	Author	Age	Sex	Symptom	Location	Size (mm)	Preoperative diagnosis	Imaging modalities	Operation	Outcome
1	2002	Umphrey et al.^6)^	51	F	RUQ pain	CBD	90	Choledochal cyst/biliary cystadenoma	CT, MRCP, ERCP	Bile duct resection	NED (4 mo)
2	2004	Shima et al.^7)^	62	F	Jaundice	CBD	41	Cystic lesion	CT, MRCP, ERCP	Bile duct resection	NED (14 mo)
3	2004	Park et al.^8)^	42	F	Jaundice	CHD	NA	Choledochal cyst	CT, MRCP, ERCP	Bile duct resection	NED (12 mo)
4	2006	Trummer et al.^4)^	52	F	NA	CBD	NA	Cholecystolithiasis with cholestasis	Intraoperative cholangiography	Enucleation	Recurrence (30 mo)
5	2010	Ray et al.^9)^	55	F	Epigastric pain, jaundice, fever	CBD-CHD	100	Hydatid cyst/biliary cystadenoma	CT	Bile duct resection	NED (3 mo)
6	2011	Hennessey and Traynor^10)^	54	F	Abdominal pain	CHD	18	Cystic tumor	CT	Bile duct resection	NED
7	2013	Watanabe et al.^11)^	31	F	Jaundice	CBD	45	Biliary cystadenoma	CT, MRCP, ERCP, EUS, IDUS, POCS	Bile duct resection	NA
8	2015	Metussin et al.^12)^	24	F	Back/RUQ pain, jaundice	CHD	35	Cystic lesion/stone	CT, ERCP	Bile duct resection	NED (36 mo)
9	2016	Safari et al.^13)^	32	F	Jaundice, weight loss	CHD	23	Stenosis (malignancy susp.)	CT, MRI, ERCP, POCS	Bile duct resection	NED (12 mo)
10	2020	Aljubran et al.^14)^	48	F	Jaundice	CBD	20	Cystic lesion/stone	CT, MRCP, ERCP	Bile duct resection	NA
11	2021	Paspala et al.^15)^	55	F	RUQ pain, jaundice, weight loss	CBD	20	Cystic lesion (malignancy susp.)	MRI, MRCP, ERCP	PPPD	NED (24 mo)
12	2023	Deng et al.^16)^	54	F	Jaundice	CBD	50	Space-occupying lesion	CT, MRCP, ERCP, POCS	Bile duct resection	NED (24 mo)
13	2024	Masaki et al.^17)^	29	F	Jaundice	CHD	30	Epithelial neoplasm/myogenic tumor susp.	CT, MRCP, POCS	Bile duct resection	NED (4 mo)
14	2025	Peña Montañez et al.^18)^	42	F	Jaundice, fever	CBD	85	Cystic lesion	MRCP	Bile duct resection	NED (3 mo)
15	2026	Present case	48	F	Jaundice, abdominal pain	CBD	60	Primary extrahepatic biliary MCN susp.	CT, MRI, MRCP, ERCP, EUS	RPPPD	NED (6 mo)

CBD, common bile duct; CHD, common hepatic duct; ERCP, endoscopic retrograde cholangiopancreatography; EUS, endoscopic utrasound; F, female; IDUS, intraductal ultrasonography; MCN, mucinous cystic neoplasm; mo, months; MRCP, magnetic resonance cholangiopancreatography; NA, not available; NED, no evidence of disease; POCS, peroral cholangioscopy; PPPD, pylorus-preserving pancreaticoduodenectomy; RPPPD, robotic pylorus-preserving pancreaticoduodenectomy; RUQ, right upper quadrant; susp., suspected

Preoperative diagnosis of primary extrahepatic biliary MCN is challenging. One reason for this is its rarity. Furthermore, due to the difficulty in differentiation using only CT or MRI, nonspecific diagnoses such as “cystic lesion” accounted for the majority of the reviewed cases, as shown in **Table 1**. Given that a definitive diagnosis strictly requires the histopathological identification of OLS, detailed multimodal imaging, particularly EUS, may help in establishing a strong preoperative suspicion of this rare entity, as this case suggests. According to the 2019 WHO Classification, imaging shows biliary MCN as a multilocular cystic mass with smaller cysts in the cyst wall, a characteristic finding often referred to as the cyst-in-cyst appearance. Malignancy is suggested by findings such as irregular thickness of the wall, internal septations, and papillary projections.^1)^ In the present case, this characteristic cyst-in-cyst feature served as a crucial clue for the preoperative clinical suspicion. Furthermore, EUS allowed for detailed evaluation of tumor morphology and extent. Since reports on EUS findings of primary extrahepatic biliary MCN are extremely rare, it is noteworthy that this case presented findings similar to those of pancreatic MCN. This suggests that the general imaging features of pancreatic MCN can be a useful reference for the preoperative suspicion of primary extrahepatic biliary MCN. Because detailed reports on the multimodal imaging of this rare entity remain exceptionally scarce, our case provides a valuable benchmark, demonstrating the effectiveness of a comprehensive imaging approach in achieving a high degree of preoperative suspicion.

Biliary MCN is generally considered not to have communication with the bile duct, which is an important point of differentiation from IPNB, which is characterized by bile duct communication.^2)^ In the present case, IPNB was excluded based on the absence of bile duct communication on ERCP, lack of intraductal papillary growth on EUS, and no evidence of mucin hypersecretion in the bile duct. Recently, POCS has been reported to be useful for differential diagnosis because it allows direct visualization of the microstructure of the tumor surface and evaluation of mucin production in the bile duct.^11,16,17)^ Watanabe et al.^11)^ successfully suspected this disease preoperatively, wherein POCS played a key role by allowing direct observation of the tumor surface. In this case, however, POCS was not performed due to the patient’s history of post-ERCP pancreatitis at the previous hospital, anticipated insertion difficulty given the lesion’s proximity to the duodenal papilla, and the strong suspicion of MCN based on characteristic EUS findings. Preoperative biopsy is generally not recommended due to the potential risk of seeding and low diagnostic yield caused by sampling error of the subepithelial OLS.^3)^ Therefore, definitive diagnosis depends on histopathological examination of the resected specimen, particularly the identification of OLS.^2)^ In this case, preoperative biopsy was not performed to avoid the potential risk of peritoneal seeding and, given the patient’s history of cholangitis, the risk of disseminating infection to the cyst. Furthermore, since the tumor showed a tendency to increase in size and MCN was strongly suspected, surgical resection was considered necessary regardless of the biopsy result. Therefore, we prioritized surgical resection without histological confirmation.

Since biliary MCN is recognized as a neoplasm with potential for invasive carcinoma, complete surgical resection is the standard of care. Extremely high recurrence rates of 90%–100% have been reported with incomplete treatments such as cyst aspiration, fenestration, and internal drainage performed in the past.^3)^ Depending on the tumor location, complete resection necessitates PD, extrahepatic bile duct resection, or hepatectomy. Tumor enucleation may also be an option, but Trummer et al.^4)^ reported a case of recurrence 30 months after enucleation of a tumor incidentally discovered during cholecystectomy, eventually requiring bile duct resection. Therefore, the indication for enucleation must be carefully judged, considering the risk of recurrence due to incomplete resection or leakage of cyst contents.^19)^ In the present case, the tumor extended to the intrapancreatic bile duct, necessitating PD. While previously reported cases were managed via open surgery, we successfully treated this case using a minimally invasive robotic approach. Regarding surgical management, this case suggests that the robotic approach can be a useful option. Specifically, its high-definition 3D magnified view and multi-articulated instruments facilitated delicate dissection under severe inflammatory adhesions caused by recurrent cholangitis. This allowed us to strictly adhere to the key oncological principles relevant to this cystic tumor: complete preservation of the tumor capsule, avoidance of content spillage, and achievement of an adequate surgical margin. To the best of our knowledge, this is the first reported case of primary extrahepatic biliary MCN treated by robotic surgery.

The prognosis after complete resection is generally good.^1)^ While malignant transformation from benign biliary MCN to invasive carcinoma has been histologically documented over long-term follow-up,^20)^ reported transformation rates ranged from 20% to 30% in past studies.^5,21)^ However, under the current definition requiring OLS, the incidence of invasive carcinoma is considered relatively low.^2)^ Also, it has been suggested that biliary MCN may have a lower malignant transformation rate compared to pancreatic MCN.^2)^ Therefore, achieving complete surgical resection is paramount for ensuring an excellent long-term prognosis.

## CONCLUSIONS

Primary extrahepatic biliary MCN is an extremely rare disease. However, this case suggests that detailed multimodal imaging, particularly EUS, may help in establishing preoperative suspicion of biliary MCN by identifying specific features such as the characteristic cyst-in-cyst appearance, internal septations, and a thick capsule. The characteristic findings presented herein will serve as a valuable reference for the preoperative differentiation of this rare entity. Complete surgical resection is the standard treatment and offers a favorable prognosis. As demonstrated in this case, robotic surgery could be a precise and minimally invasive option. However, considering this is a single case report with a short follow-up period of 6 months, whether it truly does not compromise oncological radicality requires careful evaluation through further case accumulation and long-term observation. To the best of our knowledge, this is the first report of robotic surgery for this condition, representing a feasible therapeutic option.

## DECLARATIONS

### Availability of data and materials

All the data generated or analyzed during this study are included in this article.
